# Reusable, Noninvasive, and Sensitive Fluorescence Enhanced ZnO-Nanorod-Based Microarrays for Quantitative Detection of AFP in Human Serum

**DOI:** 10.1155/2021/9916909

**Published:** 2021-07-15

**Authors:** Saima Rafique, Farukh Kiyani, Sumbal Jawaid, Rubina Nasir, Mahmoosh Ahmad, Shazia Bashir, Muhammad Idress, Jan Sher Khan, Rizwan Akram

**Affiliations:** ^1^Department of Physics, Air University, PAF Complex, E-9, Islamabad 44000, Pakistan; ^2^Department of Animal Sciences, Quaid e Azam University, Islamabad 44000, Pakistan; ^3^Department of Biomedical Engineering and Technology, University of Engineering and Technology, Taxila, 47080 Punjab, Pakistan; ^4^Department of Physics and Applied Mathematics, Pakistan Institute of Engineering and Applied Sciences, Islamabad 45650, Pakistan; ^5^Department of Biosciences, University of Wah, Wah Cantt, Pakistan

## Abstract

The fabrication of sensitive protein microarrays such as PCR used in DNA microarray is challenging due to lack of signal amplification. The development of microarrays is utilized to improve the sensitivity and limitations of detection towards primal cancer detection. The sensitivity is enhanced by the use of ZnO-nanorods and is investigated as a substrate which enhance the florescent signal to diagnose the hepatocellular carcinoma (HCC) at early stages. The substrate for deposition of ZnO-nanorods is prepared by the conventional chemical bath deposition method. The resultant highly dense ZnO-nanorods enhance the fluorescent signal 7.2 times as compared to the substrate without ZnO-nanorods. The microarray showed sensitivity of 1504.7 ng ml^−1^ and limit of detection of 0.1 pg ml^−1^ in wide dynamic range of 0.05 pg-10 *μ*g ml^−1^ for alpha fetoprotein (AFP) detection in 10% human serum. This immunoassay was successfully applied for human serum samples to detect tumor marker with good recoveries. The ZnO-nanorod substrate is a simple protein microarray which showed a great promise for developing a low-cost, sensitive, and high-throughput protein assay platform for several applications in both fundamental research and clinical diagnosis.

## 1. Introduction

Liver is a vital body organ involved in the detoxification of metabolites, protein synthesis, and production of biochemical which are important for digestion. Hepatocellular carcinoma (HCC) or liver cancer is the sixth most commonly occurring cancer in the world [1]. Approximately, 85% of the liver cancer is detected in Asian and African countries, whereas Europe and America have low ratio of liver cancer [2, 3]. In Pakistan, liver cancer is the fifth most commonly occurring cancer in men and ninth most commonly occurring in women, and Pakistan also lies in the top twenty five countries list whose population is facing the liver cancer [4]. The treatment of liver cancer is being done according to the stage on which it is diagnosed [5]. The diagnosis research focuses more on targeted therapy in order to reduce risk of damaging or killing the normal cells. Most commonly used therapy is the angiogenesis inhibitor usage to cure the cancer [6, 7].

Alpha fetoproteins (AFP) are the proteins being present in the serum of the liver, and their level rises in the patients with liver cancer; they are measured as a tumor marker and as a result of the effective treatment of the tumor, its level is being reduced in the serum [8]. An elevated level of AFP is being reported in about 70% of the people being diagnosed with the primary liver cancer. Hence, it is the key material that is being tested when we are going to know about the stage of the liver cancer [9]. AFP value from normal to >100,000 ng ml^−1^ can be produced by HCC [10]. However AFP levels more than 400 ng ml^−1^ in a high-risk patient are diagnostic of HCC [11]. Therefore, the identification of early HCC is important for surveillance of patients.

The patients being diagnosed with HCC at early stage has survival rate of 31% but in the late stage, the survival rate is 11% [12]. At early stages, the blood test is performed which includes estimation of alpha fetoprotein in serum. In order to make diagnosis, it is essential to develop the nanoparticles due to their capability to detect cancer in early stages. For example, Li et al. incorporated ZnO nanoparticles for formation of hybrid microarray hexadecyltrimethoxysilane-ZnO nanoparticles-aminopropyltriethoxysilane (HDS-ZnO-APS) [13]. They observed that incorporation of ZnO nanoparticles leads towards the enhanced fluorescence signal with dynamic range of 0.01 to 10 ng ml^−1^ for detection of human IgG. Beside this, Adalsteinssan et al. used ZnO nanoscale arrays for ultrasensitive detection of cytokine in urine [14]. They concluded that the ZnO nanorod array-based approach is highly beneficial for early detection of disease. Most importantly, Dorfman et al. reported the use of ZnO nanoscale material for enhancement in fluorescence detection of protein [15]. They established four types of microarrays: (i) direct ZnO nanorods, (ii) stripped ZnO arrays (ii), open square arrays, (iv) and filled square arrays, with physical adsorption of protein on microarrays. They observed enhanced fluorescence detection due to the presence of ZnO nanomaterial as no fluorescence emission was observed from the same biomolecules that were adsorbed elsewhere on the substrate. Pal et al. developed an ultrasensitive enzyme-linked immunosobent assay (ELISA) for carcinoembryonic antigen (CEA) using ZnO nanoparticles [16]. They showed that a threefold enhanced chemiluminescence signal is found by use of ZnO nanoparticles compared to conventional ELISA. Zhao et al. use ZnO nanorod arrays and in situ zeoliticimidazolate framework-8 (ZIF-8) coating for detection of carcinoembryonic antigen [17]. They investigated fluorescence enhancement characteristic of the ZIF-8 towards organic fluorescence and successfully applied it to protein marker detection. They obtained a limit of detection (LOD) for CEA as low as 0.01 pg ml^−1^, and the dynamic range was 0.01 pg ml^−1^ to 100 pg ml^−1^. Thus, nanoparticles are paving the way for diagnostic tools especially for early diagnosis, more individualized treatment options, and better therapeutic success rates.

In this paper, a simple and cost-effective ZnO-nanorod microarray was developed for the detection of the liver cancer to achieve the enhancement in detection, sensitivity, and reusability for the primal cancer detection. During fabrication of fluorescence microarray, the essential objective was (a) to develop a substrate, (b) enhancement in fluorescence signal, (c) attachment of antibodies, and (d) reusability of microarray. The microarrays were made on the glycidoxypropyltrimethoxysilane (GPTS) and ZnO-nanorod substrate. ZnO-nanorods have been found to provide better fluorescent signal performance as compared to the substrate without ZnO-nanorods. The high surface area and change in density of photonic mode of ZnO-nanorods offer sensitive detection of AFP biomarker and AFP in human serum samples. Therefore, a novel reusable ZnO-nanorod grown substrate has been developed which detect AFP in human serum and demonstrate comparative lower detection limits without use of any amplification tag.

## 2. Experiment

### 2.1. ZnO-Nanorod Synthesis

The synthesis was performed by the chemical bath deposition method. It required a glass slide (25 mm × 75 mm × 1 mm) which was cleaned ultrasonically with ethanol and deionized water. Then, the cleaned glass slide was soaked in the 30 ml (5 mM) solution of potassium permanganate (KMnO_4_) for half an hour. After that, the glass slide was rigorously washed with deionized water and then dipped slightly tilted in the solution of 0.1 M zinc nitrate hexahydrate, 4% (v/v) of ammonium hydroxide, and ethanolamine10% (v/v). Finally, the solution was heated up to 75°C for 30 minutes [18]. The slide with developed ZnO-nanorods was washed with deionized water and dried under stream of nitrogen.

### 2.2. Synthesis of Antibody Microarrays

**Figure 1:**
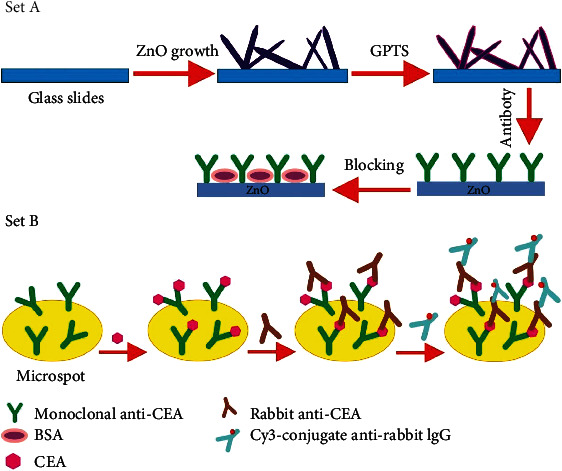
Schematic illustration of different steps for preparation of protein microarray in step A and detetcion of different concentrations of alpha fetoprotein biomarker in step B.

For the fabrication of antibody microarrays, ZnO-nanorod slide was incubated in the 3-glycidoxypropyl trimethoxysilane (GPTS) ethanol solution for about two hours. This would be followed heating at 110°C for about two hours in vacuum as illustrated by Figure 1 (step A). Then, the antibody microarrays were developed on the ZnO-nanorod slide with the help of a robotic spotter known as the VersArray chip writer™ Figure 1 (step B). The printed slide was incubated for about 8 hours at room temperature. The printed buffer contains 250 *μ*g ml^−1^ of monoclonal antibody (anti AFP) in phosphate buffer saline (PBS, 0.01 M) which also has 2.5% glycerol Triton X-100 (0.004%). After that, 20 mg ml^−1^ bovine serum albumin (BSA) solution was used to block remaining sites on the ZnO-nanorods in order to remove the nonspecified binding of the antibodies. Finally, the tris buffered saline (TBS) 0.05 M solution was used for rigorous washing Figure 1 (step B). A simple GPTS-modified (without ZnO-nanorods) glass slide was also printed using the same 50 *μ*g mL^−1^ quantity of Cy3-labeled anti-goat IgG in 0.01 M PBS containing 2.5% glycerol and 0.004% Triton X-100 as the ink for comparison purposes, the fluorescent microarrays as shown in Figure 1 (step B).

The repeatability of experimental measurement of the calibration curve was studied at AFP concentration of 0.1, 10, 100, and 1000 ng ml^−1^. Four anti-AFP spots were printed on single ZnO-nanorod glass slides by microarray printer. Next, different concentrations of AFP were used on this printed spot (four concentrations on single glass slide), and experiment was repeated five days in a row to check the sensitivity of calibration curve.

### 2.3. Real Sample Analysis

In order to perform serum analysis, the antibody microarrays were first incubated with the sample solution having 10% human blood serum for approximately 2 hours. The serum samples were obtained from NORI Cancer Hospital, Islamabad, Pakistan. The serum samples were diluted (10 time) with 0.1 M PBS. The microarrays were then rinse with the tris buffered saline (TBS) for 1 hour and were allowed to react with the rabbit anti-AFP in the blood serum which acts as the recognition antibody. Next, the Cy3-labeled anti rabbit IgG of 2 *μ*g ml^−1^ quantity was allowed to react for half an hour as illustrated in Figure 1 (step B). Finally, the slide was washed with the tris buffered solution (TBS) and deionized water and dried at 37°C. With the optimized conditions, the obtained matrix matched calibration curve (MMCC) was compared with the standard AFP calibration curve. Two-way ANOVA was used to compare the MMCC and standard calibration curve for each sample. A SPSS21 (statistical software) with the Wilcoxon signal ranked test (WSRT) was used to analyze the comparison data of proposed method and ELFA. The *p* value at >0.05 was considered no significant difference with confidence limit of 94%. Recovery test was also performed by spiking different concentrations of AFP on human serum samples.

### 2.4. Characterization

The surface morphology images were measured and collected by the field emission scanning electron microscope (FESEM) FESEM and JEOLEM-2100F system. X-ray diffraction (XRD) patterns were examined using an advanced X′Pert Pro X-ray diffractometer (Panalytical Xpert Pro) which was operated at a voltage of 40 kV with the help of Cu K*α* radiation. The pro scanner-ray microarray scanner (Bio-Rad, USA) at 543 nm excitation was used to obtain the fluorescent images. AFM images are obtained using tapping mode of Agilent's PicoPlus AFM. The probe type is silicon nitride tip having a spring constant of 0.437 N/m and tip radius of 10 nm. To detect the grains, the watershed algorithm was used using Gwyddion software version 2.58. For roughness parameters, root-mean-squared roughness (RMS) was used to quantitatively describe the topography of surfaces.

## 3. Results and Discussion

### 3.1. Surface Morphology

**Figure 2:**
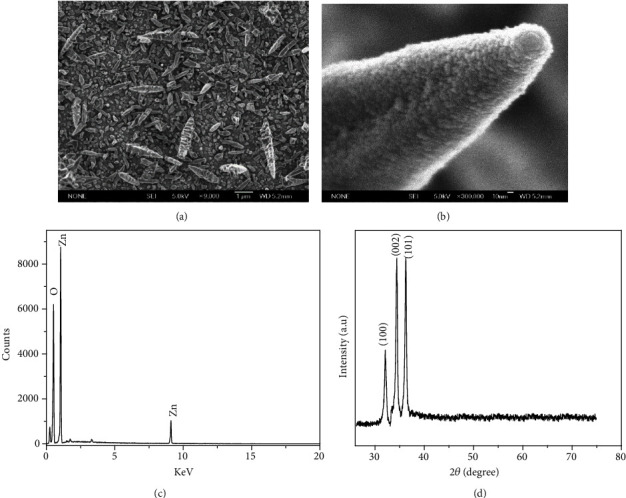
(a, b) SEM images, (c) the EDX spectra, and (d) the XRD pattern obtained for the ZnO-nanorods grown on glass slide by the chemical bath method.

The glass slide was treated with KMnO_4_ solution which results in formation of Mn-hydroxide deposit on the slide. This deposit acts as a seed layer that allows growth of ZnO-nanorods [19]. The morphology is strongly dependent on the activation conditions such as KMnO_4_ concentration, temperature, time, and reducing agent [20]. Figures 2(a) and 2(b) represent the morphology of prepared ZnO-nanorods. The ZnO-nanorods are well deposited with random orientations. The ZnO-nanorods have varied diameters from 21.61 ± 2.04 to 55.81 ± 4.65 nm and hexagonal faceted structures with sharp tips. The sharp tip was due to the synthesis of ZnO-nanorods at low temperatures (<85°C). At low temperature, the film deposited has poorly defined shape and small crystal formation at the start of deposition. The better well-defined hexagonal and vertically aligned ZnO-nanorods can be obtained at higher temperature [21].The density of ZnO-nanorods is also good and very uniform across larger area (Figure 2(a)) which is vital for fabrication of antibody microarrays with low array-to-array and spot-to-spot detection variations, but there is large variation in length of ZnO-nanorods. This was due to the random orientation of initially deposited ZnO at the start of growth. This causes termination of growth of some of the rods compared to the rods growing perpendicularly [22]. This mechanism generates the coalescence of ZnO crystal on surface of larger rods as can be seen in Figure 2(a).  Figure 2(c) shows the EDX of the ZnO-nanorod samples. In spectra, the prominent peak at 0.52 KeV, 1.03 eV, and 9 KeV was distinctly observed. Out of all these peaks, the X-ray energies of 0.52 KeVand1.03 eV represent the emission from *K* shell of oxygen and *L* shell of zinc, respectively. The additional X-ray energy at 9 KeV represents emission from Zn core levels [23]. Overall, the occurrence of these basic Zn and O emissions endorses the existence of Zn and O atoms in prepared ZnO-nanorods.

Figure 2(d) shows the XRD of the ZnO-nanorods grown by chemical bath method. The (1 0 0), (0 0 2), and (1 0 1) peaks of ZnO were clearly observed while (0 0 2) and (1 0 1) peaks exhibit almost same intensity. The sharp ZnO peak at (0 0 2) shows the hexagonal wurtzite structure with preferred orientation along *c*-axis, whereas the peak assigned (1 0 1) suggests the oriented ZnO-nanorods on the glass substrate [24]. These results elucidate that due to the random orientation of initially deposited ZnO at the start of growth as confirmed from FESEM produce highly crystalline ZnO-nanorods but with an angle on substrate.

### 3.2. Optimization

#### 3.2.1. Effect of ZnO-Nanorods on Fluorescent Intensity

**Figure 3:**
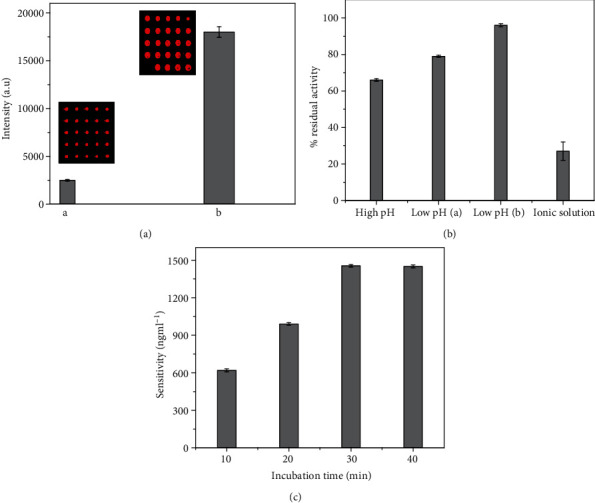
Efficiency of fluorescence microarray towards detection of AFP. (a) The effect on fluorescence signal by synthesizing ZnO-nanorods on substrate. (b) Effect of different types of regeneration solution on residual activity. (c) The effect of incubation time towards detection of AFP.

The effect of ZnO-nanorods on fluorescent intensity was investigated by using cy3-labeled anti-goat IgG. To investigate this effect, two types of substrate were prepared one with GPTS and another with ZnO-nanorod modified glass slide. The spots obtained for both the surfaces are shown in Figure 3(a). The GPTS modified glass slide showed quasicircular spots with dimension around 98 *μ*m and fluorescent intensity of 2500 ± 89 a.u. (shown in inset of Figure 3(a0). The spots obtained for the ZnO-nanorod modified surface were uniform in size with mean diameter of 260 *μ*m, whereas drastic increase in fluorescent intensity from 2500 ± 89 a.u to 18000 ± 819 a.u was observed. The observed enhancement in florescence is related to the change in density of photonic mode of ZnO-nanorods [25]. Hence, this suggests that the spots with high intensity and uniform size were obtained from ZnO-nanorod modified glass slide which can be helpful for wide dynamic range detection of alpha fetoprotein.

#### 3.2.2. Regeneration Solutions

In order to break the binding between anti-AFP and AFP, different regeneration solutions were used. For this purpose, three classes of solutions are as follows: (1) high pH (20 mM NaOH, pH = 12.0), (2) low pH, [{10 mMHCl, pH = 2.0: 25 mM (low pH (a)) and glycine–HCl, pH = 2.5 (low pH (b))}], and (3) ionic solution 2 M NaCl. The efficiency of regeneration solution was evaluated by residual activity percentage (RA%) = 100% × (Δ*I*_1_/Δ*I*_2_), where Δ*I*_1_ is the change in signal intensity after regeneration and Δ*I*_2_ represents the change after binding of antibody and antigen before regeneration. It can be observed in Figure 3(b) that the residual activity falls sharply at more acid or alkaline pH values. This could be due to loss of activity or destruction of modified immunoassay at high, low pH, and for ionic solutions. However, use of glycine-HCl retained high value of % RA. This suggests that developed microarray provides reliable outcome with % RA greater than 95%. So, glycine–HCl solution will be used to examine the cycles of regeneration.

#### 3.2.3. Incubation Time

The effect of time of incubation on sensitivity of microarray at concentration of 100, 10, 1, and 0.1 pg ml^−1^ was also examined and shown in Figure 3(c). It can be observed that the sensitivity increases with the increase in incubation time but it saturates at higher incubation time. As the analyte is detected within minutes after incubation and then saturates, therefore, 30 min was selected as time of incubation for determination of tumor biomarker from solution. This was to ensure the prevention of saturation and for the better performance of microarrays.

### 3.3. Analytical Performance

#### 3.3.1. Linear Dynamic Range, Limit of Detection, and Limit of Quantification

**Figure 4:**
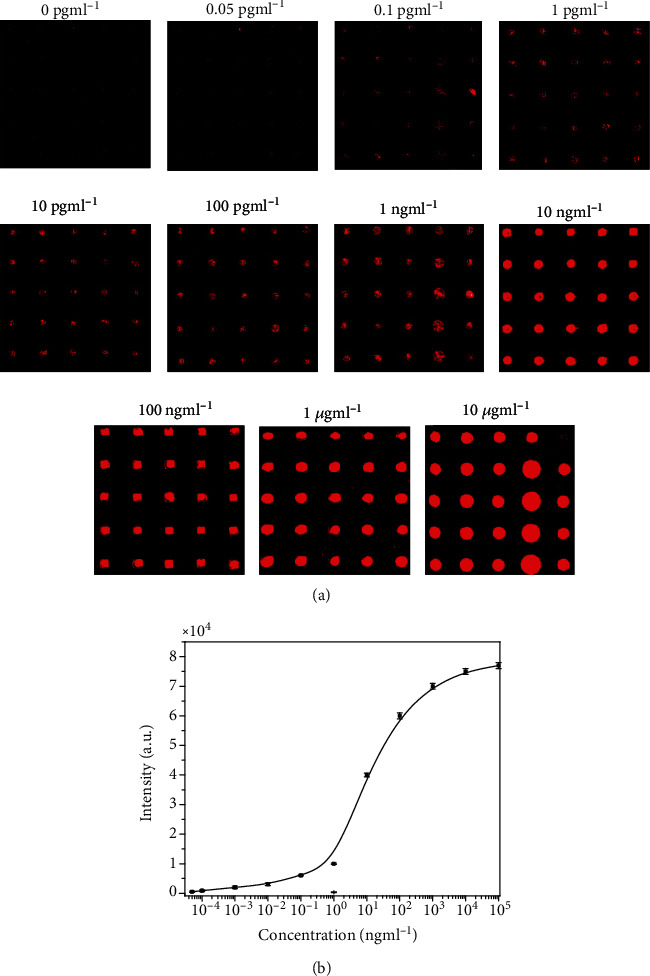
Microarray slide containing different concentrations of AFP ranges from 0 pg ml^−1^ to 10 *μ*g ml^−1^ AFP antigen solutions followed with cy3-labeled anti-rabbit IgG. (b) Calibration curve of microarray at different concentrations of AFP.

Alfa fetoprotein (AFP) is a glycoprotein produced by the fetal liver, and serum AFP levels are often elevated in hepatocellular carcinoma (HCC) [26]. So, AFP is an indicative protein for HCC and other chronic liver diseases. In present work, different concentrations of AFP were tested to find the lowest concentration of AFP levels to examine the performance of protein microarray in 10% human serum with AFP as an analyte. Various concentrations of AFP range from 0.05 pg ml^−1^ to 10 *μ*g ml^−1^ that were applied to each subarray and incubated for 1 hour at room temperature. The 0.01 M phosphate buffer solution (PBS) containing 10% human serum (v/v) was used as a negative control sample. After incubation with recognition antibody and Cy3-labeled secondary antibody, the slides were rigorously washed and then scanned. The florescent images obtained for different concentrations are shown in Figure 4(a). It showed that the signal intensity increased with increase in concentration. The signal strength obtained at the lowest concentration demonstrates clearly the attachment of antibody on ZnO-nanorod modified glass slide. This attachment is strong enough to sustain even after successive washing. It can be observed in Figure 4(a) that negative control also shows weak fluorescent signal. This can be due to the damaging of ZnO-nanorods during microarray printing [27].The dose response curve was plotted between the fluorescent intensity and logarithm of concentration as shown in Figure 4(b). The dynamic range covers 6 orders from 0.05 pg ml^−1^to 10 *μ*g ml^−1^ and limit of detection (LOD) of 0.1 pg ml^−1^. The LOD was determined as 3 × SD/sensitivity and the limit of quantifications from 10SD/sensitivity in which SD was the standard deviation of blank microarray. The sensitivity was obtained from linear fit of linear region in the dose-response curve. The different parameters obtained for protein microarray are listed in Table 1. It can be seen from Table 1 that a wide dynamic range from 0.05 pg ml^−1^to 10 *μ*g ml^−1^ and limit of detection of about 0.1 pg ml^−1^ were obtained for the ZnO-nanorod modified surface. This value is better compared to the metal-linked immunosorbent assay (MeLISA) [28]. However, the prepared protein microarrays showed better performance using ZnO substrate compared to directly AFP modified protein microarrays [29], fluorescence protein microarray for detecting serum AFP in patients with HCC [30], and polydopamine nanosphere@silver nanoclusters (PDAN@AgNCs) system for multiplexed detection [31]. It can be concluded from here that the cut-off AFP value for early-stage HCC was 17.4 ng ml^−1^from clinical screening in blood samples [32]. So, with ZnO-nanorods microarray, 1000 times diluted samples can be detected efficiently.

#### 3.3.2. Reproducibility

To investigate the reproducibility of ZnO-nanorod-based microarray chip, three different microarray chips were prepared under the same experimental condition (AFP concentration was fixed to 10 pg ml^−1^), and measurements were performed on five different days on the same chip. The fluorescent signal was obtained with (*n* = 10, *m* = 3), where *n* is the number of measuring points on a microarray chip and *m* was the number of measurements on single microarray. The reproducibility was confirmed by evaluation of interassay chip variation and interday variation. The interassay variance coefficient (CV) is shown in Table 2. The interassay variance was found 1.9% which showed as good reproducibility. Similar results were obtained for interday variance coefficient.

#### 3.3.3. Reusability

**Figure 5:**
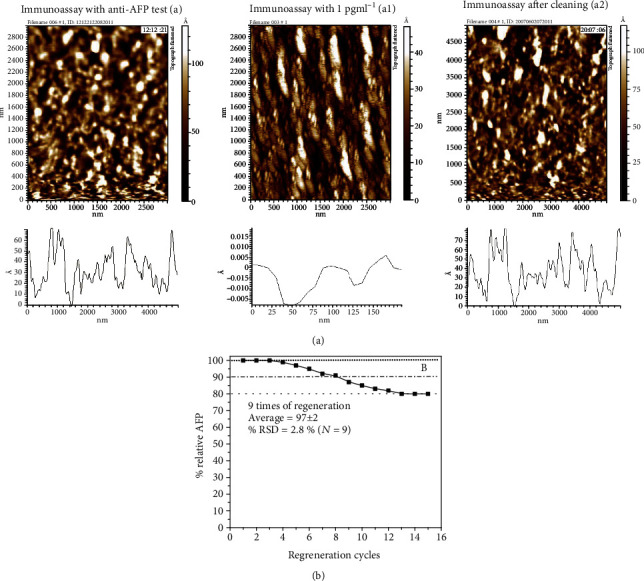
Surface topography images of (a) after immobilization of anti-AFP (a1) after attachment of AFP (a2) microarray after use of regeneration solution. (b) Relationship between the percentage change in fluorescence signal and cycles of regeneration.

To investigate the reusability, a regeneration solution (25 mM glycine–HCl, pH = 2.5) for 20 min was used after detecting antigens of concentrations 100, 10, 1, and 0.1 pg ml^−1^. The surface morphology was studied with atomic force microscopy (AFM); moreover, the relative change in the fluorescence intensity response was examined for immunoassay. The surface topography of immunoassay with anti-AFP, after immobilization of AFP and 5 cycles of regeneration is shown in Figure 5(a), (a1), and (a2), shows the 2D topography images obtained from AFM.

The immunoassay with monoclonal anti-AFP has irregular protrusion with the average grain size of 135 ± 7 nm with the RMS value of 50.3 ± 4.2 nm. When AFP solution having concentration 1 pg ml^−1^ was added on immunoassay, surface structures with many bundles separated by gaps are observed with the RMS value changed to 1.12 ± 0.15 nm. These bundles were due to the fact that functional group (OH) interacts with each other. So, every OH group participates in hydrogen bonding, either in small clusters or chains consisting of a large number of OH groups. This change in surface morphology and roughness compared to immunoassay with anti-AFP showed that AFP connects the surface. After 5 cycles of regeneration, the antigens bound to the electrodes were found to be removed, as the granular surface with the average grain size of 157 ± 4 nm again appeared with the average surface roughness 55.8 ± 2.1 nm which is close to the roughness profile before the regeneration. This illustrated that before and after cleaning of substrate, there is no chemical immobilization that is on the surface of immunoassay.

Similarly, the relative Δ*I* (intensity change) was examined, and it was observed that it remains stable for 7 cycles of regenerations with average response of 97 ± 2 and % RSD of 2.8 as shown in Figure 5(b).With the increase in regeneration cycles, the intensity response decreased and losts below 90%. This shows that due to the damage of surface, the response decreased. This suggests that the proposed immunoassay could be reused for further applications, and it will be valuable for clinical applications.

#### 3.3.4. Interference

**Figure 6:**
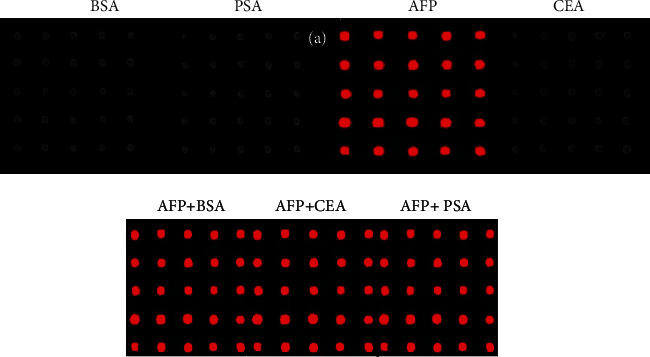
Responses of immunoassay for detetcion of AFP with different interferences and comparison of analyte with mixture of analytes.

To examine the reliability of immunoassay, the microarray spotted with AFP was measured with interfering analytes, specifically, prostate-specific antigen (PSA, 100 ng ml^−1^), carcinoembryonic antigen (CEA, 100 ng ml^−1^), and alpha fetoprotein (AFP, 100 ng ml^−1^). After reaction with cy3-labeled anti-goat IgG for one hour, the ZnO-nanorod modified slide was fluorescent image, and results are shown in Figure 6(a). It is observed that the subarray on the microarray reacting with its corresponding antibody exhibits bright fluorescent signal. There was no considerable difference between AFP and AFP mixed with different analytes that was observed. This suggests that the resulting microarray exhibits an excellent specificity. It is also observed that there is small variation in spot size and florescent signal. The spot-to-spot variation is small with the relative standard deviation less than 5%. This is due to the homogenous printing of microspots on randomly oriented ZnO-nanorod substrate [33]. This shows that the prepared immunoassay exhibits excellent specificity.

#### 3.3.5. Analysis of Real Samples

A comparative study was performed to validate the performance of fluorescent-based immunoassay under optimal conditions. The immunoassay matrix effect was investigated on the human serum samples. The sensitivity, one with standard tumor marker and other serum samples spiked with standard, was performed by two-way ANOVA. There was no considerable difference that was observed at confidence limit of 95% (*p* > 0.05). It shows that matrix does not affect the response of the system. Initially, the serum samples were diluted with PBS, and results obtained are shown in Table 3. The Wilcoxon signal rank test (WSRT) was employed to compare the concentration of tumor marker from the proposed method and reference values obtained from hospital by ELFA. The relative standard deviation was 0.34–1.6%, showing no considerable difference between two methods. These results indicate the potential applicability of fluorescence immunoassay towards clinical diagnosis. Next, the recoveries of AFP were evaluated in ten human serum samples. The three different concentrations of antigen (1, 10, 100 ng ml^−1^) were spiked in human serum, and results obtained are shown in Table 4. The obtained recoveries are in the range of 93.4–99.02% with relative standard deviation of 0.7–1.9%. These results are consistent and provide accurate results for serum samples.

## 4. Conclusions

In summary, the experiments demonstrated that engineered nanoscale ZnO-nanorods can serve as ideal substrates for identifying and screening protein–protein interaction. The protein microarray was fabricated using ZnO-nanorods synthesized by the chemical bath method. Results reveal that a significant enhancement was observed in fluorescent signal by ZnO-nanorod synthesis. The antibody microarray showed a low limit of detection of 0.1 pg ml^−1^ and wide dynamic range of 0.05 pg-10 *μ*g ml^−1^ which is 6 orders of magnitude without using conventional costly methods. The proposed microarray shows good accuracy, reproducibility, and specificity in blood serum samples. Moreover, the results obtained from microarray were compared with results of ELFA, and no considerable difference was observed in two methods. Since the fabrication of ZnO-nanorod-based microarrays is scalable, reusable, and inexpensive technique, it offers great potential for a new type of high performance high-throughput protein microarrays for disease biomarker screening in early diagnosis.

## Figures and Tables

**Table 1:** Performance parameters of prepared antibody microarray for AFP detection.

LOD pg ml^−1^	Dynamic range	Linear equation	*R* ^2^	Sensitivity ng ml^−1^
0.1	0.05 pg-10 *μ*g ml^−1^	1504.7 log(*x*) + 2399.8	0.9565	1504.7

**Table 2:** Interassay variation coefficient result for AFP concentration 10 pg ml^−1^.

Standard samples, *N* = 3	Fluorescent intensity (mean)	Standard deviation of mean	% CV of mean
Low signal	3576	187	2.2
High signal	3987	247	1.7

**Table 3:** Comparison results of microarray (*n* = 5) and the conventional method (ELFA method) for the detection of AFP inhuman serum samples.

	AFP (ng ml^−1^)	RSD (%)	Ref.
	Found		
Human serum 1	10.24 ± 0.07	1.02	10.24
Human serum 2	—	—	—
Human serum 3	173.59 ± 0.05	0.07	173.55
Human serum 4	220.00 ± 0.01	0.34	223.00
Human serum 5	420.10 ± 0.04	1.6	420.80
Human serum 6	351.81 ± 0.03	0.82	352.22
Human serum 7	—	—	—
Human serum 8	—	—	—
Human serum 9	922.22 ± 0.7	0.8	920.00
Human serum 10	8.87 ± 0.01	0.97	8.88

**Table 4:** The average recoveries (*n* = 5) with the relative standard deviations of AFP in human serum samples.

	Spiked (ng ml^−1^)	AFP	RSD (%)
		Recovery (%)	
Human serum 1	1	96.12 ± 0.07	0.81
	10	96.28 ± 0.21	2.20
	100	95.10 ± 0.21	3.10

Human serum 2	1	97.75 ± 0.04	0.70
	10	97.91 ± 0.02	1.90
	100	98.59 ± 0.06	0.79

Human serum 3	1	96.30 ± 0.10	1.20
	10	95.80 ± 0.10	1.50
	100	96.10 ± 0.30	1.90

Human serum 4	1	97.49 ± 0.02	0.30
	10	95.68 ± 0.07	0.77
	100	96.10 ± 0.30	2.00

Human serum 5	1	97.50 ± 0.06	4.40
	10	93.40 ± 0.05	0.85
	100	95.22 ± 0.09	3.30

Human serum 6	1	94.10 ± 0.40	3.20
	10	93.40 ± 0.05	0.40
	100	94.20 ± 0.40	6.00

Human serum 7	1	99.02 ± 0.08	1.02
	10	95.90 ± 0.20	3.90
	100	96.50 ± 0.08	4.60

Human serum 8	1	93.36 ± 0.07	1.03
	10	97.40 ± 0.09	1.37
	100	97.40 ± 0.09	0.79

Human serum 9	1	97.63 ± 0.05	1.40
	10	96.80 ± 0.50	0.77
	100	98.65 ± 0.05	2.00

Human serum 10	1	96.11 ± 0.20	3.00
	10	96.51 ± 0.06	0.87
	100	97.23 ± 0.10	0.71

## Data Availability

The data that support the findings of this study are available from the corresponding author upon reasonable request.
